# Effect of Hydroxyvalerate Molar Percentage on Physicochemical and Degradation Properties of Electrospun Poly(3-hydroxybutyrate-co-3-hydroxyvalerate) Fibrous Membranes and Potential Application for Air Filtration

**DOI:** 10.3390/polym17202719

**Published:** 2025-10-10

**Authors:** Yaohui Liu, Cheng-Hao Lee, Yanming Wang, Chi-Wai Kan, Xiao-Ying Lu

**Affiliations:** 1 Department of Construction Environment and Engineering, Technological and Higher Education Institute of Hong Kong, Tsing Yi, New Territories, Hong Kong, Chinaxylu@thei.edu.hk (X.-Y.L.); 2 School of Fashion and Textiles, The Hong Kong Polytechnic University, Hung Hom, Kowloon, Hong Kong, China

**Keywords:** poly(3-hydroxybutyrate-co-3-hydroxyvalerate) (PHBV), electrospinning, fibrous membrane, air filtration, soil degradation

## Abstract

This study investigates the air filtration capabilities of fibrous membranes fabricated via electrospinning, with a focus on optimizing processing parameters. Specifically, Poly(3-hydroxybutyrate-co-3-hydroxyvalerate) (PHBV), a well-characterized biodegradable polyester, was electrospun to produce membranes exhibiting precisely controlled surface microstructures. The optimal fiber morphology was attained under conditions of a 20 kV applied electric field, a solution flow rate of 0.5 mL·h^−1^, a polymer concentration of 13 wt.%, and a needle inner diameter of 0.21 mm. The microstructural features of the electrospun PHBV membranes were characterized using scanning electron microscopy (SEM). Complementary analysis via ^13^C nuclear magnetic resonance (NMR) spectroscopy confirmed that the membranes comprised pure 3-hydroxyvalerate (3HV) copolymerized with 3-hydroxybutyrate (3HB) terminal units, with 3HV mole fractions ranging from 17% to 50%. The incorporation of different molar percentages of 3HV in PHBV membrane significantly enhances its durability, as evidenced by Ball Burst Strength (BBS) measurements, with an elongation at burst that is 65–86% greater than that of ASTM F2100 level 3 mask. The nanofibrous membranes exhibited a controlled pore size distribution, indicating their potential suitability for air filtration applications. Particle filtration efficiency (PFE) assessments under standard atmospheric pressure conditions showed that the optimized electrospun PHBV membranes achieved filtration efficiencies exceeding 98%. Additionally, the influence of 3HV content on biodegradation behavior was evaluated through soil burial tests conducted over 90 days. Results indicated that membranes with lower 3HV content (17 mol.%) experienced the greatest weight loss, suggesting accelerated degradation in natural soil environments.

## 1. Introduction

Electrospinning is a versatile and efficient technique for producing fibrous membranes with diameters ranging from micrometers to nanometers. These membranes possess a unique combination of properties—high surface-area-to-volume ratio, high porosity, interconnected pore structures, and controllable surface chemistry—making them exceptionally suitable for environmental remediation [1,2]. For example, highly porous fibers of different polymers and bead-on-string heterostructured fibers with distinctive morphology have been achieved by electrospinning techniques [3,4]. The high-performance nanofiber membrane filter prepared by electrospinning technology could enhance the filtration efficiency with low pressure drop compared to commercial air filters [5,6,7,8]. However, due to good anti-corrosion ability, most synthetic polymer electrospun materials are nondegradable in the natural environment, causing secondary pollution to the environment. On the contrary, biodegradable polymers with a wide range of sources and economic benefits can not only theoretically solve environmental issues such as microplastic pollution but can also handle the conundrum of resource scarcities. Therefore, biodegradable electrospun nanofiber membranes offer the added advantage of reducing secondary plastic pollution and have attracted more attention in the field of air filtration and aligned with the principles of an eco-friendly economy [2,9,10,11].

Polyhydroxyalkanoates (PHAs) are naturally occurring polyesters composed of hydroxyalkanoate (HA) monomers, synthesized and stored intracellularly by various bacterial species as a reserve of energy in conditions of excess carbon sources and/or limited nutrients [12,13,14,15,16]. PHAs are recognized as biodegradable plastics, exhibiting mechanical properties comparable to those of traditional thermoplastics, such as polyethylene (PE) and polypropylene (PP), thereby serving as viable alternatives to petroleum-based polymers in commercial applications [17,18,19,20]. The incorporation of plasticizers and low molecular weight additives has been identified as an effective strategy to enhance the flexibility and processing characteristics of polyhydroxybutyrate (PHB) [20]. These plasticizers infiltrate the polymer matrix, leading to a reduction in deformation tension, elastic modulus, and viscosity, which in turn increases the flexibility of the polymer chains, enhances fracture resistance, and modifies the dielectric constant [20]. The flexibility of PHB can also be improved through the introduction of HA comonomers, such as 3-hydroxyvalerate (3HV), resulting in the formation of the copolymer poly-3-hydroxybutyrate-co-3-hydroxyvalerate (PHBV). In comparison to PHB, PHBV exhibits lower crystallinity, which contributes to its increased flexibility, enhanced impact resistance, and overall toughness, making it particularly suitable for applications in flexible packaging [6,20]. The performance characteristics of PHBV are significantly influenced by its 3HV content; as identified by Fourier Transform Infrared (FTIR) spectroscopy [21], an increase in the content of 3-hydroxyvalerate (3HV) results in a decrease in the crystallinity of the polymer, an improvement in its flexibility, and a reduction in its biodegradability [18,20]. It is particularly noteworthy that augmenting the 3HV percentage in poly(3-hydroxybutyrate-co-3-hydroxyvalerate) (PHBV) from 0% to 50% can significantly lower its melting point (T_m_) [17].

Among various bio-based plastics, polyhydroxyalkanoates (PHAs) are increasingly acknowledged as a sustainable alternative to conventional petroleum-based plastics. PHAs represent a category of bio-based, biodegradable and non-toxic polymers that exhibit thermoplastic characteristics [21,22,23] and are produced naturally by bacterial fermentation [24]. A prominent member of the PHA family is poly(hydroxybutyrate-co-hydroxyvalerate) (PHBV), which can be sourced from renewable plant materials [25,26,27]. PHBV is classified as recyclable and industrially compostable, making it suitable for application in injection molding applications. The mechanical recycling of PHBV can be conducted up to six times, depending on the concentration of 3-hydroxyvalerate (HV) within the polymer [28]. Prior studies have indicated that bio-based polyesters such as PHBV undergo natural degradation in various environments. Research has examined the enzymatic degradation of PHBV at molecular level [29,30], wherein enzymes are utilized as biocatalysts. Additionally, degradation processes involving fungi in soil [31] and in seawater and compost have also been explored [32,33]. In natural settings, PHBV exhibits a weight loss of approximately 60% after six weeks in aquatic environments, and a biodegradation rate exceeding 70% after 18 weeks in soil. Consequently, the remarkable properties of PHBV position it as a promising candidate for significant applications in environmental protection [34,35]. However, the practical application of PHBV is hindered by its inherent brittleness and suboptimal mechanical properties.

The objective of this research is to (i) investigate electrospun poly(3-hydroxybutyrate-co-3-hydroxyvalerate) (PHBV) fibrous membrane with varying percentages of hydroxyvalerate (HV), (ii) observe surface morphology of fibrous membrane, (iii) specific surface area and pore size of the membrane, (iv) analyze the chemical structure of PHBV, (v) assess its mechanical properties, (vi) evaluate its thermal and degradation characteristics, and (vii) examine its air filtration capability. The findings will elucidate the variations in the properties of PHBV during a three-month operational period in a pilot plant setting.

## 2. Materials and Methods

### 2.1. Electrospinning Process

Poly(3-hydroxybutyrate-co-3-hydroxyvalerate) (PHBV), characterized by varying molar percentages of 3-hydroxyvalerate units (17%, 20%, 25%, and 50%) and a molecular weight of 30,000 kDa (Cheng Jin Plastics Co., Ltd., Dongguan, China), was prepared in a homogeneous solution by dissolving the polymer in chloroform (GR grade, 99.8% purity, RCI Labscan Ltd., Bangkok, Thailand). Prior to the electrospinning process, the solution was transferred into a 50 mL glass syringe equipped with a metal luer lock tip (SAMCO Co., Ltd., London, UK). In accordance with our previous research, the electrospinning parameters were optimized, which included an applied voltage of 20 kV, a solution concentration of 13 wt.%, a flow rate of 0.5 mL/h, and a tip inner diameter of 0.21 mm. The distance between the needle tip and the cylindrical collector was maintained at 16 cm. These optimized parameters facilitated the establishment of a stable system, wherein the rate of fluid being forced into droplets was equivalent to the average rate at which the fluid was ejected by the jet. The polymer solutions were contained within a standard 50 mL syringe (SAMCO Co., Ltd.) and were dispensed using an NE-300 single syringe pump (Model TL-F6, Tong Li Tech. Co., Ltd., Shenzhen, China). A metal syringe needle (SAMCO Co., Ltd.) was affixed to the syringe, with the needle tips being ground to create a blunt end, thereby enhancing the stability of the Taylor cone prior to utilization. The needle tip and the rotating cylindrical collector were connected to a high-voltage power supply (Model TL-Pro, Tong Li Tech Co., Ltd., Shenzhen, China), capable of delivering a positive direct current voltage of up to 30 kV.

### 2.2. Fiber Morphology

Electrospun fibers were subjected to a thin conductive gold coating via a sputtering coater (E-1010, Hitachi Co., Tokyo, Japan), and their morphological characteristics were examined utilizing scanning electron microscopy (SEM) (TESCAN MIRA LMS, Brno, Czech Republic) at an accelerating voltage of 15 kV. The fiber diameters were quantified through SEM imaging at a magnification of 1000×, employing Image J software 1.54k for analysis.

### 2.3. Ball Burst Test

The mechanical properties of the electrospun PBHV fibrous membrane and ASTM F2100 level 3 mask [36] were assessed through ball burst testing utilizing the INSTRON 4411 Tensile Compression Testing Machine (Instron Corporation, Norwood, MA, USA). An electrospun fibrous membrane, with an area of 13 cm × 12 cm and a thickness of 0.14 mm ± 0.04 mm, along with a level 3 mask, was securely clamped between two metal rings for subsequent testing in accordance with ASTM standard D3787-07 [37]. Sandpaper was affixed to the metal rings during the ball burst testing to mitigate any slippage of the mesh. The data obtained from each sample were systematically recorded in terms of Load (Newtons) versus elongation (mm).

### 2.4. Soil Burial Degradation Test

A study on soil burial degradation under natural conditions was conducted at the campus of the Hong Kong Polytechnic University. Prior to the burial, the samples were dried in an oven at 50 °C and weighed. Multiple groups of samples were subsequently buried underneath the soil surface at 15 cm depth for a duration of 90 days. The parameters of soil temperature, humidity, and pH were primarily influenced by the natural degradation process, with the microorganisms involved originating from the indigenous microbial community present in the soil. Weight loss during the soil burial degradation was assessed at intervals of 10 days. The soil was meticulously removed using water, dried in an oven at 50 °C until a constant weight was achieved, and then weighed. The percentage of weight loss (*W_L_*) for each sample undergoing degradation was evaluated using the following equation [38,39,40]:

$$W_{L}=\;\frac{M_{b}-M_{a}}{M_{b}}\;\times 100\%$$

where *M_b_* and *M_a_* are the weights of the sample before and after degradation, respectively.

### 2.5. Thermal Analysis

Differential scanning calorimetry (DSC) was conducted on all electrospun PHBV samples, each weighing 4–5 mg, utilizing Perkin Elmer aluminum pans. The analysis was performed with a DSC 8000 (Perkin Elmer, Waltham, MA, USA) equipped with refrigerated cooling under a nitrogen atmosphere at a flow rate of 50 mL/min. Calibration of the DSC was carried out using indium and sapphire standards in accordance with the manufacturer’s protocols. The samples were initially equilibrated at 30 °C for a duration of 3 min, followed by cooling to 0 °C at a rate of 15 °C/min, and subsequently heated to 400 °C at the same rate.

The melting temperature (T_m_) and melting enthalpy (Δ*H_f_*) were ascertained from the inflection point and endothermic peak observed during the heating scan, respectively. The degree of crystallinity (*X_c_*) was calculated using Equation (1).

(1) $$X_{c}=\frac{{∆H}_{f}}{{∆H}_{f}^{o}}\times 100$$

In this study, Δ*H_f_* represents the melting enthalpy of the sample, while ${∆H}_{f}^{o}$ denotes the melting enthalpy of the pure crystalline polymer, specifically 146 J/g for poly(3-hydroxybutyrate-co-3-hydroxyvalerate) (PHBV) [41]. The data were analyzed utilizing Perkin Elmer Differential Scanning Calorimetry (DSC) software, Pyris software version 11. The thermal stability of the purified PHBV was assessed through thermogravimetric analysis (TGA) conducted with a Perkin-Elmer TGA-7 instrument (PerkinElmer, Shelton, CT, USA). Duplicate samples weighing 4–5 mg were subjected to heating from 30 °C to 500 °C at a rate of 20 °C/min in a nitrogen atmosphere. The analysis of the data was performed using Pyris Manager v13.3 software.

### 2.6. Qualitative ^13^C Nuclear Magnetic Resonances

Electrospun PHBV samples were solubilized in CDCl_3_, and ^13^C-nuclear magnetic resonance (NMR) spectra were obtained using an Avance Bruker 400 MHz spectrometer (Bruker Biospin AG., Fällanden, Switzerland) at ambient temperature. The resulting spectra were analyzed with Mestrelab Mnova software v16 (Barcelona, Spain). Additionally, the ^13^C-NMR spectra facilitated the assessment of the sequence distribution of 3HV and 3HB in PHBV, as previously documented in the literature [18,42,43].

### 2.7. Filtration Measurement

The filtration efficiency of particulate matter (PMs) was evaluated using a PMs gas particulate matter detector (HYX-MS600-6, Eranntex Co., Shenzhen, China). The air flow rate was maintained at 85 L/min, with a test area of 3.2 cm^2^ and air resistance of 12.5 Pa.

### 2.8. Brunauer–Emmett–Teller (BET) Surface Area Analysis

Brunauer–Emmett–Teller (BET) surface area [44] and pore size distribution were determined from nitrogen adsorption isotherm data at 77 °K (liquid nitrogen temperature) using BET surface area and a porosimetry analyzer (Micromeritics ASAP 2460, Micromeritics Instrument Corporation, Norcross, GA, USA). Prior to measurement, fibrous membrane specimens (0.15–0.17 g) were degassed at 120 °C for 8 h under nitrogen atmosphere. BET surface areas were determined from 9-point adsorption isotherms that were completed using a 0.06–0.2 relative pressure range (P/P_0_). Pore-size and the distributions were calculated from Barrett–Joyner–Halenda (BJH) [45] desorption data in 0.02–0.99 relative pressure range (P/P_0_).

## 3. Results

### 3.1. Qualitative ^13^C Nuclear Magnetic Resonances

**Figure 1:**
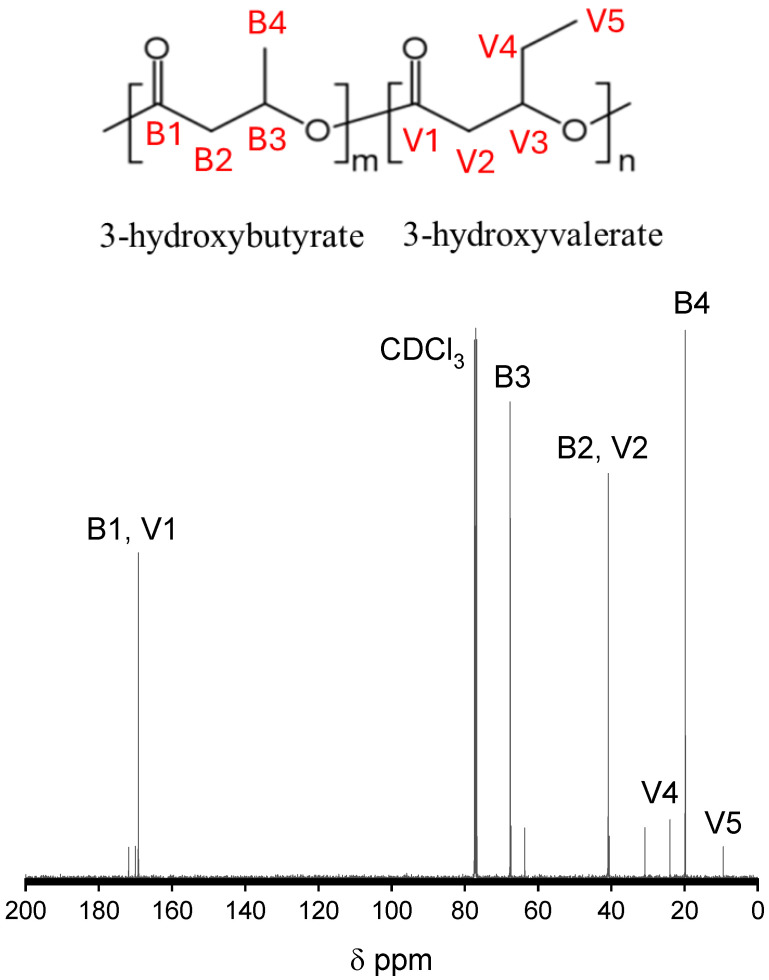
^13^C NMR of electrospun PHBV membrane.

Figure 1 presents the resonance ^13^C-spectra associated with the electrospun polymers analyzed in this study. The resonance peaks observed align closely with those reported in existing literature [43,44,45,46]. In the ^13^C-spectrum of polyhydroxybutyrate (PHB), four distinct peaks are identifiable: the carbonyl peak (B1) at 169.17 ppm, the methyl carbon peak (B4) at 19.79 ppm, and the methylene (B2) and methine (B3) carbon peaks at 40.81 ppm and 67.63 ppm, respectively. These peaks are major characteristics of polyhydroxybutyrate (PHB). In contrast, the ^13^C-spectrum of polyhydroxybutyrate-co-hydroxyvalerate (PHBV) reflects the copolymeric nature of PHB and hydroxyvalerate (HV). The methylene groups from both HB and HV contribute to the peaks observed between 26 and 40 ppm, with carbonyl peaks appearing around 169 ppm [43,44,47]. These signals correspond to the diad structure of HB and HV units, where B denotes butyrate and V denotes valerate. The peak at 169.17 ppm is attributed to the carbonyl resonance of PHB and is attributed to the BB sequence. As noted by Doi et al. [48], intermediate peaks of lower intensity are associated with either BV or VB units at the end groups of PHB. The peaks identified within the methylene (-CH_2_-) group are consistent with those documented in the literature. Notably, the peak at 40.81 ppm, corresponding to the methylene group of the HB unit (B2), displays a shoulder indicative of two overlapping peaks, attributed to the diad sequences BV and BB. For the HV unit, the signals from the side-chain methylene groups at the side chain (V4) and the main-chain (V2) can be distinguished, although both signals may split into four peaks with nearly equivalent intensities. The main-chain methylene (V2) signal is observed within the range of 40.54–40.91 ppm, while the side-chain methylene (V4) exhibits a shift around 23.96 ppm.

The characterization of the PHBV samples, specifically to ascertain whether they exhibited random or simple block-copolymer sequences, was conducted using ^13^C-NMR spectroscopy, as illustrated in Figure 1. This analysis was based on the examination of diad and triad sequences [18]. The carbon resonance peaks corresponding to HB and HV were observed to be split into multiplets, a phenomenon attributed to the presence of diad and/or HV-centered triad comonomer sequences [18,42]. The carbonyl region displayed three distinct peaks at δ = 169.17, 169.25, and 169.40 ppm, which were assigned to various diad sequences: 3HB*3HB, 3HV*3HB, and 3HV*3HV, respectively. Notably, the resonance associated with the main chain carbon linked to the 3HV side-chain methylene carbon (V3) was neither detected nor did it exhibit splitting into any triad sequences. Within the PHBV samples, certain signals were identified as corresponding to carbonyl and methylene carbon groups (B2, V2, V4), with a specific signal for methylene carbon (B2) also being observed.

### 3.2. Surface Morphology of Fibrous Membrane

**Figure 2:**
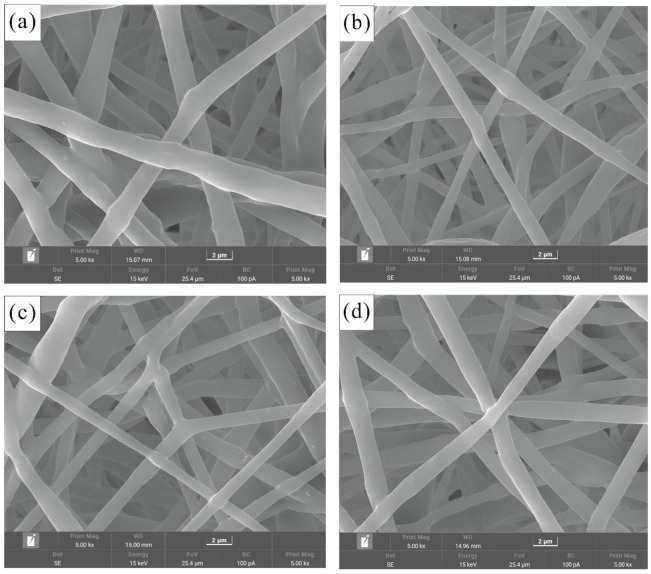
SEM images of the electrospun PHBV nanofibers prepared with (**a**) 50% HV; (**b**) 25% HV; (**c**) 20% HV (**d**) 17% HV in chloroform.

Following optimization, all PHBV fibrous membranes exhibited smooth and inter-fused fibers, devoid of beads, with a mean fiber diameter of 2.2 ± 0.3 μm (see Figure 2). This finding aligns with the morphological observations previously documented by Figueroa-Lopez et al. [49] and Melendez-Rodriguez et al. [50] concerning the same polymer. Notably, a reduction in the mole percentage of HV in the electrospun PHBV fibrous membrane resulted in a smooth fiber morphology characterized by a narrow diameter distribution and the absence of beads, yielding an average fiber diameter of 1.86 ± 0.72 μm.

### 3.3. Thermal Properties of Poly(3-hydroxybutyrate-co-3-hydroxyvalerate) (PHBV)

**Figure 3:**
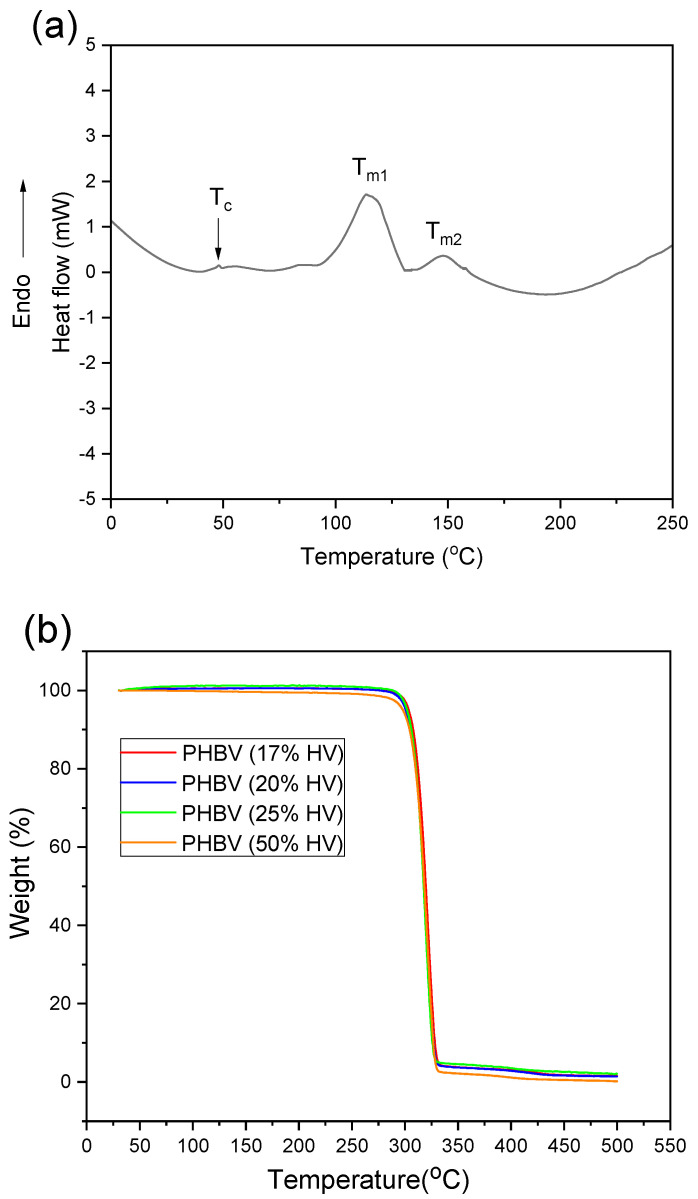
(**a**) DSC profile of the representative electrospun PHBV fibrous membrane (50 mol. % HV) exhibited glass transition temperature (T_c_), 1st melting temperature (T_m1_) and 2nd melting temperature (T_m2_), respectively and (**b**) TGA profile of PHBV fibrous membrane with various HV mol. %.

The electrospun PHBV fibrous membranes were subjected to differential scanning calorimetry (DSC) to assess their thermal characteristics, including melting temperature (T_m_), crystallization temperature (T_c_), and degree of crystallization (*X_c_*) in Table 1. The reanalyzed data are presented in Table 1. The observed melting temperatures T_m1_ and T_m2_ for the typical PHBV sample as shown in Figure 3a was consistent with the findings presented by Guho et al. [51]. The phenomenon of isomorphism may account for the occurrence of two distinct melting peaks [51,52]. Given that PHBV exhibits a semi-crystalline structure, crystals with a higher hydroxyvalerate (HV) percentage are characterized by a relatively high proportion of the amorphous phase, resulting in their melting (T_m1_) at lower temperatures. Conversely, PHBV domain with a lower HV content possess a higher degree of crystallinity, leading to melting at elevated temperatures (T_m2_) [52,53]. Additional factors contributing to the presence of two melting points include variations in crystalline morphologies (such as perfection, multiple polymorphs, recrystallization), differences in the molecular weight distribution of crystallites, aging and relaxation effect of the amorphous phase [52,53].

The crystallization temperature (Tc) of the electrospun PHBV membranes varied between 54 °C and 79 °C (Table 1). Sample containing 17 mol.% HV, exhibited the highest Tc at 78.6 °C, whereas the PHBV sample with 50 mol.% HV showed the lowest Tc of 54.6 °C. There was a negative linear correlation between crystallization temperature (T_c_) and 3HV mole percentage. An increase in 3HV content results in an increase in the amorphous phase and require less time and energy to achieve whole crystallization process which leads to a reduction in crystallinity (X_c_) and crystallization temperature (T_c_) [54,55]. A lower T_c_ indicates that the complete crystallization process will take a longer time [54].

Thermogravimetric analysis (TGA) was conducted to assess the thermal degradation characteristics of the purified poly(3-hydroxybutyrate-co-3-hydroxyvalerate) (PHBV) samples, as detailed in Table 2. All electrospun PHBV samples exhibited a uniform one-step degradation profile as illustrated in Figure 3. The TGA curves facilitated the determination of the temperature at which maximum weight loss occurred (T_d_). The degradation temperature (T_d_) values for the electrospun PHBV fibers ranged from 318 °C to 325 °C, which is notably higher than the values documented in existing literature for PHBV (T_d_ = 286 °C) [56] and poly(3-hydroxybutyrate) (PHB) (T_d_ = 280 °C) [57]. It is noteworthy that thermal degradation of PHB initiates just above its melting temperature (T_m_), which constrains its process conditions [17]. The incorporation of 3-hydroxyvalerate (3HV) in PHBV results in a reduction of T_m_ relative to T_d_, thereby widening the processing window in applications like molding and extrusion processes [52,54,58,59]. The thermal degradation of PHBV is characterized by a singular process attributed to a non-radical random chain scission mechanism involving a six-membered ring transition state [52,57,60,61]. The onset degradation temperature was observed from 266.72 °C to 274.94 °C, while the offset degradation temperature ranged from 298.72 °C to 302.48 °C. These temperature values are marginally elevated compared to those reported in other studies [19,62]. Specifically, an onset temperature of 177–238 °C, T_d_ of 276–296 °C, and an offset temperature range of 291–309 °C have been documented for PHBV containing 5–20% 3HV [19]. Additionally, Samorì et al. reported that T_d_ (1%) (the temperature at which 1% weight loss) was approximately 258 °C and a degradation temperature (T_d_) of 284 °C for PHB [62], implying an enhancement in the thermal stability of the PHBV examined in this study relative to pure PHB. It was also observed that T_d_ is inversely proportional to 3HV percentage in PHBV copolymer, further contributing to the thermal stability of the PHBV fibrous membranes [17].

### 3.4. Ball Burst Strength (BBS)

**Figure 4:**
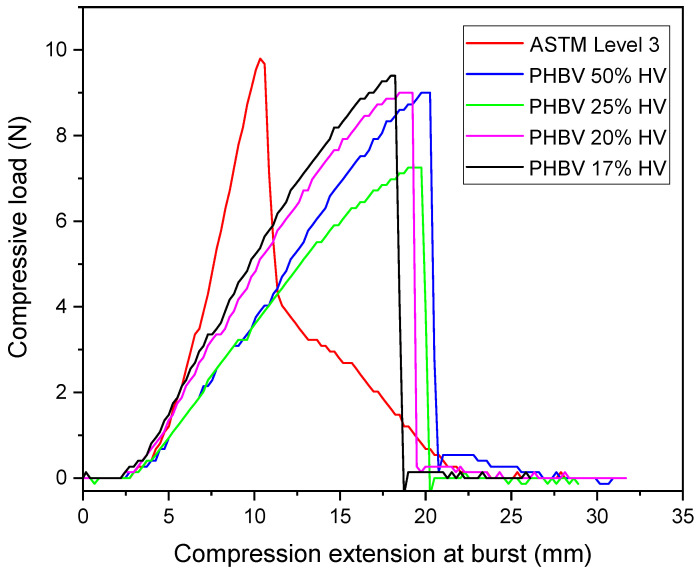
Ball burst profile (compressive load vs. elongation at burst) of PHBV fibrous membrane and commercial ASTM F2100 level 3 mask.

The results pertaining to ball burst strength and elongation are illustrated in Figure 4 and detailed in Table 3. The PHBV membrane containing 17% HV demonstrated a maximum compressive load of 9.34 N, which is comparable to the 9.71 N load value associated with ASTM F2100 level 3 masks. The elongation at burst for level 3 mask was recorded at 11.0 mm, whereas the PHBV fibrous membrane exhibited a significantly elongation at burst of 18.21 mm. In contrast, the PHBV variant with a higher HV content (50 mol.% HV) displayed a lower compressive load of 8.86 N, while achieving a maximum elongation at burst of 20.05 mm. When considering membranes of similar thickness, it is evident that the electrospun PHBV fibrous membrane demonstrates an acceptable compressive load in comparison to commercially available surgical masks. The results from the ball burst test indicate an inverse relationship between compressive strength and elongation for electrospun PHBV fibrous membranes with varying HV content. Upon the application of force, the electrospun fibrous membrane experiences partial rupture, allowing for elongation that exceeds that of ASTM level 3 mask, attributed to highly uniform fiber diameters [63,64].

### 3.5. Filtration Efficiency

In the context of practical applications, the electrospun PHBV fibrous membranes require a reference fabric or membrane for the assessment of their filtration properties. This study utilized a commercially available non-biodegradable polypropylene (PP) melt-blown membrane as the reference material. The particle filtration efficiencies of all electrospun membranes were evaluated while maintaining a consistent average thickness of 0.14 mm ± 0.04 mm, which was determined by the spinning duration. Table 4 presents the filtration efficiency of the electrospun PHBV fibrous membrane as a function of HV mol.%. As illustrated in Table 4, the particle filtration efficiency of the ASTM F2100 level 3 polypropylene-based mask was determined to be 98.23% for particle diameter range from 0.3 mm to 10 mm, thereby meeting the requisite filtration standards. In contrast, the filtration efficiency of the electrospun PHBV membrane, with HV content from 17 mol.% to 50 mol.%, was measured in the range between 98.7% and 99.1%. According to the GB 19083-2010 standard [65], the inhalation resistance of the mask at a gas flow rate of 85 L/min should not exceed 343.2 Pa. Notably, the electrospun PHBV fibrous membrane demonstrated air resistance of only 12.5 Pa, indicating its efficacy as a protective filtration material [66,67,68,69].

The electrospun membrane, characterized by elongated and linear fibers, demonstrates a notable pore size when subjected to particles measuring 0.3 µm. Conversely, the average surface pore diameter of these straight fibers is expected to fall within the submicron range (less than 1 mm), which aligns with the dimensions of the particles in question. Consequently, the pores present on the fiber surface are likely capable of capturing test particles ranging from 0.3 µm to 10 µm. Furthermore, there exists a strong correlation between the specific surface area of the filter and its pore size. Filters constructed from fibers with smaller diameters may exhibit enhanced particle collection efficiency, primarily due to the particle diffusion mechanism [66]. The implementation of a filter with diminished pore sizes in its internal layers can markedly influence the direct entrapment of larger particles. According to the molecular sieve effect, for optimal particle capture, the dimensions of the particle must be equal to or greater than the average pore size of the fibrous membrane. Furthermore, a textured fiber surface may enhance the friction coefficient, thereby promoting the accumulation of particles [70].

The sieve effect posits that the particle filtration efficiency of electrospun PHBV fibrous membranes can reach nearly 100% for micron-sized particles and exceed 95% for submicron-sized aerosol particles. This efficiency is attributed to the membrane’s structural characteristics, particularly its relatively high fiber stacking density, which is effective in capturing larger particles while demonstrating limitations in filtering particles ranging from 0.3 μm to 10 μm in size [69,71].

### 3.6. Specific Surface Area and Pore Size Distribution

BET method was used for determination of specific surface areas of fibrous membranes or for observation of changes that appear in the structure during modification of organic-based nanofibers [72]. Significant information has been disclosed indicating that the BET method was employed in the characterization of fibrous membranes with internal porosity [73] or in investigation of the influence of the thermal-cooling cycle on the subsequent structure of fibrous membrane [74]. The relationship of specific surface area as a function of average pore diameter is shown in Figure 4. The BET surface area increased with a decreasing pore diameter. N_2_-physisorption of the fibers showed a significant reduction in the average pore diameter from 10.251 nm (untreated) to 6.745 nm (3 thermal-cooling cycles) (Table 5). The increased specific surface area was expected because the specific surface area typically has inversely correlation with pore volume. The specific surface area depends strongly on the fiber size, pore volume, and pore size distribution. The results indicate that an annealing temperature of 100 °C, above the Tg of PHBV, was high enough to induce microstructural changes in the fibers on the micro- and meso-scales.

### 3.7. Degradation of Electrospun Fibrous Membrane in Soil

**Figure 5:**
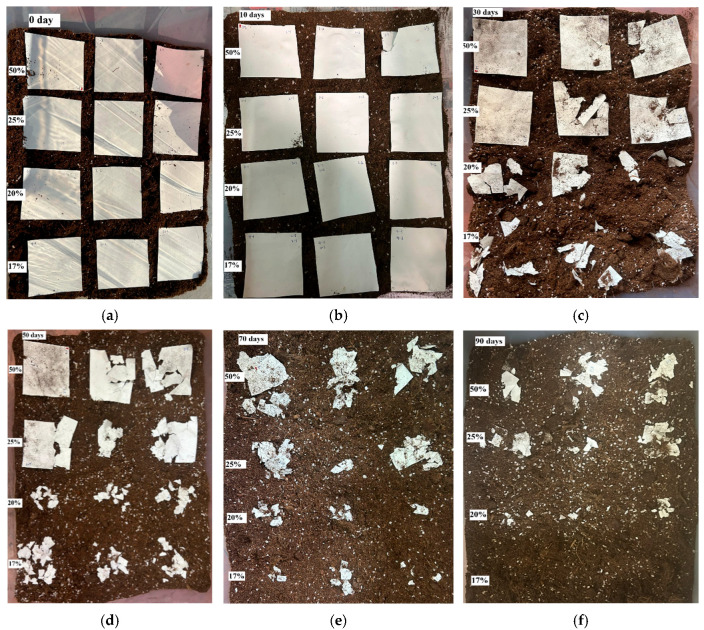
Optical images of the electrospun PHBV fibrous membrane under soil burial test at (**a**) 0 day, (**b**) 10 days, (**c**) 30 days, (**d**) 50 days, (**e**) 70 days and (**f**) 90 days, respectively.

Figure 5 illustrates that following a 90-day period of outdoor soil burial, the water loss (WL) of the multi-scale structured membrane reached 100%. This phenomenon can be attributed to the increased interface between water and microorganisms present in the soil, which is facilitated by the extensive fibrous surface area of the membrane. Furthermore, the major degradation products of poly(3-hydroxybutyrate-co-3-hydroxyvalerate) (PHBV) are carbon dioxide (CO_2_) and water (H_2_O), which can be assimilated by plants through the process of photosynthesis, thereby establishing a recyclable and sustainable cycle [57,58]. Consequently, the utilization of PHBV as a raw material holds promise for mitigating environmental pollution and resource wastage associated with the disposal of conventional masks. This advancement is pivotal for the promotion of environmentally friendly and sustainable mask alternatives, contributing significantly to the overall health and sustainability of the environment.

**Figure 6:**
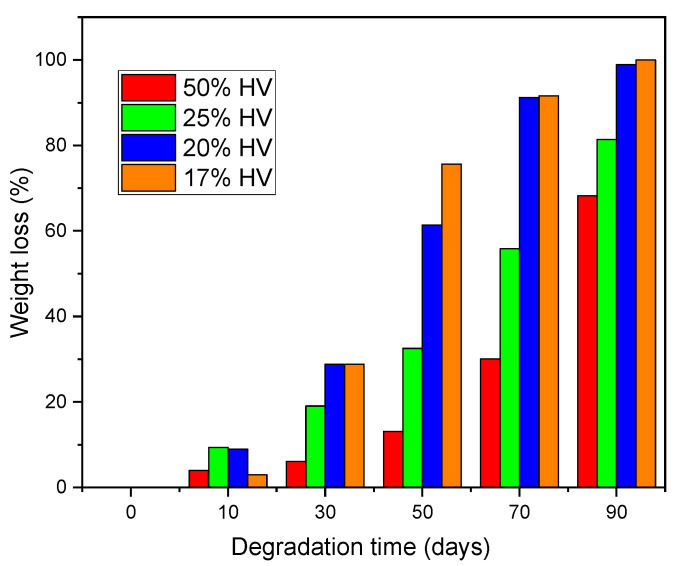
Weight loss (%) of PHBV samples with various HV mole percentages vs. degradation time in soil environment.

The assessment of weight loss serves as a crucial indicator of the bulk degradation of PHBV films containing varying mole percentages of HV. Figure 6 illustrates the weight loss profile of electrospun PHBV fibrous membranes in relation to degradation time over a duration of 100 days. Measurements of weight loss were halted after this period, as hydrolytic degradation led to the fragmentation of the membrane into exceedingly small debris. Consequently, the residual fragments could not be accurately quantified for the purpose of determining the weight loss of the PHBV membrane.

The degradation analysis indicated that the highest weight loss was observed in the PHBV film containing 17 mol.% HV, in contrast to the film with 50 mol.% HV. After a degradation period of 70 days, the PHBV films with 17 mol.% and 20 mol.% HV exhibited weight losses of approximately 91.2% and 91.6%, respectively. In comparison, the film with 50 mol.% HV demonstrated a weight loss of 30%, while the film with 25 mol.% HV exhibited a weight loss of 56%. These findings suggest that the HV percentage in PHBV copolymer significantly affects the degree of degradation.

## 4. Conclusions

The objective of this study was to investigate the thermal, mechanical and air filtration characteristics of poly(3-hydroxybutyrate-co-3-hydroxyvalerate) (PHBV) with varying hydroxyvalerate (HV) content. Additionally, the degradation behavior of PHBV with different HV compositions in a soil environment over a period of 90 days was examined. The results of weight loss analysis indicated that the molar percentage of HV in the PHBV copolymer significantly affects the degradation rate of PHBV in a natural environment. It was observed that the degradation of the PHBV film was relatively slow during the initial 10 days, followed by a rapid degradation phase after 30 days. The highest percentage of weight loss in the copolymer was recorded for PHBV membranes with lower HV content (17 mol.%). The preliminary gradual degradation of PHBV, succeeded by a steady degradation rate within the temperature range of 14 to 21 °C, indicates that PHBV-based biomaterials may exhibit stability for an estimated duration of two weeks prior to degradation in physiological conditions. This degradation profile positions PHBV as a suitable material for air filtration masks with short-term stability.

The current study examines the impact of varying concentrations of 3-hydroxyvalerate (3HV) (17%, 20%, 25%, and 50% mol) on the processability and final characteristics of electrospun films derived from P(3HB-co-3HV). The findings indicate that the incorporation of different molar percentages of 3HV into the PHBV membrane significantly enhances its durability, as evidenced by an elongation at burst that is 65–86% greater than that of masks classified at ASTM F2100 level 3.

This study elucidates the characteristics of copolymers with varying 3HV content and presents innovative strategies to address the challenges associated with low processability and ductility, thereby facilitating the development of a potentially biodegradable material suitable for air filtration applications. In comparison to commercially available ASTM F2100 Level 3 masks, the multiscale electrospun membrane demonstrates superior filtration efficiency (exceeding 95% for PM 0.3). Notably, in contrast to conventional masks, the resultant filter exhibits biodegradable properties, with the multiscale structured fibrous membranes fully decomposing after more than 90 days of burial in outdoor soil. This research introduces a compelling methodology for the fabrication of biodegradable mask filters, and it is posited that such filters could serve as a viable alternative to current commercial masks in future endeavors aimed at environmental sustainability and carbon neutrality.

## Figures and Tables

**Table 1:** DSC profile of PHBV with various HV mole percentages: 1st melting temperature (T_m1_), 2nd melting temperature (T_m2_).

PHBV (%HV)	T_m1_ (°C)	T_m2_ (°C)	T_c_ (°C)	*X_c_* (%)
50%	120.52	141.74	54.56	23.37
25%	121.02	143.86	58.39	25.96
20%	121.07	146.84	63.53	33.62
17%	121.18	147.31	78.55	50.71

**Table 2:** TGA profile of PHBV with various HV mole percentages: onset temperature (T_onset_) and offset temperature (T_offset_) of the degradation process and degradation temperature of weight loss (T_d_).

PHBV (%HV)	T_d_ (°C)	T_onset_ (°C)	T_offset_ (°C)
50%	287.86	266.72	298.72
25%	296.07	276.76	303.93
20%	296.11	274.54	301.62
17%	296.85	274.94	302.48

**Table 3:** Ball burst test (compressive load vs. elongation at burst) of electrospun PHBV membrane and ASTM F2100 level 3 polypropylene (PP) mask.

	Maximum Compressive Load (N)	Compression Elongation at Burst (mm)
ASTM F2100 level 3 mask	9.71	11.00
PHBV 50% HV	8.86	20.05
PHBV 25% HV	7.39	19.32
PHBV 20% HV	9.27	19.13
PHBV 17% HV	9.34	18.21

**Table 4:** Particle filtration efficiency (PFE %) of electrospun PHBV membrane with various HV mol.% and ASTM F2100 level 3 PP mask.

Particle Diameter (μm)	ASTM F2100 Level 3	PHBV (50% HV)	PHBV (25% HV)	PHBV (20% HV)	PHBV (17% HV)
10	100.000	100.000	100.000	100.000	100.000
5.0	100.000	100.000	100.000	100.000	100.000
2.5	99.999	99.999	99.999	99.999	99.999
1.0	99.999	99.999	99.999	99.999	99.999
0.5	100.000	96.321	96.688	96.4848	97.378
0.3	89.588	95.691	96.422	96.193	97.367
PFE(%)	98.231	98.668	98.851	98.753	99.124

**Table 5:** The specific surface area (SSA) and average pore diameter of electrospun fibers obtained from different HV mol.%. (25 °C → 100 °C → 25 °C) denotes one heating-cooling cycle.

Electrospun Membrane with HV mol.%	BET Surface Area (m^2^/g)	Average Pore Diameter (nm)
untreated	0.927	10.251
One heating-cooling cycle	2.343	7.124
Three heating-cooling cycles	2.692	6.745

## Data Availability

Data are contained within the article.
